# *Trechus* from Ethiopia with Aedeagus Right Side Superior in Repose, an Unusual Character State in Trechine Beetles (Coleoptera: Carabidae)

**DOI:** 10.3390/insects16030328

**Published:** 2025-03-20

**Authors:** Joachim Schmidt, Yeshitla Merene, Yitbarek Woldehawariat, Arnaud Faille

**Affiliations:** 1 General and Systematic Zoology, University of Rostock, 18055 Rostock, Germany; 2 Department of Zoological Sciences, Addis Ababa University, Addis Ababa P.O. Box 1176, Ethiopia; merene73et@yahoo.ca (Y.M.); yitbarek.woldehawariat@aau.edu.et (Y.W.); 3 Amhara Agricultural Research Institute, Bahir Dar P.O. Box 527, Ethiopia; 4 Department of Entomology, Stuttgart State Museum of Natural History, 70191 Stuttgart, Germany; arnaud.faille@smns-bw.de

**Keywords:** *Abyssinotus*, Afroalpine, inverse male genitalia, new species, systematics, Trechini

## Abstract

The Miocene volcano Mt. Choke in northern Ethiopia is known for its very species-rich *Trechus* fauna. In addition, the enormous morphological diversity that is expressed within a single subgenus, *Abyssinotus*, which is endemic to northern Ethiopia, is unique within the global *Trechus* fauna. In this paper, we describe eight additional new species and three subspecies of the *Trechus* subgenus *Abyssinotus*, all of which are endemic to Mt. Choke. These species belong to two different lineages within *Abyssinotus*, each differing from other lineages of the subgenus by certain morphological character states described in this paper and alternatively named the *T. lobeliae* and the *T. basilewskianus* subgroups. Several species of these subgroups are characterised by an apomorphical inverse male genital, with the right side up in repose. The *T. lobeliae* subgroup comprises two species and is monomorphic with respect to this character. The *T. basilewskianus* subgroup comprises eight species, including six species with monomorphically inverse male genitalia and two species with male genitalia monomorphically in the ‘normal’ position. These are the first examples within Trechinae in which inverse male genitalia are a species- or even group-specific characteristic. A brief summary of the distribution of this trait within the Carabidae is provided. We also present a new identification key for the *Trechus* species known so far from Mt. Choke.

## Simple Summary

The Ethiopian highlands are a hotspot of taxonomic and morphological diversity for the ground beetle genus *Trechus*. The subgenus *Abyssinotus* is particularly noteworthy in this respect. This article describes eight new species and three new subspecies endemic to the Miocene volcano Mt. Choke in northern Ethiopia. These species belong to two subgroups, referred to as the *T. lobeliae* and the *T. basilewskianus* subgroups. The peculiarity is that most of the species in these subgroups are characterised by male genitalia rotated 180° in the abdomen in the resting position compared with the normal position. This means that the original ventral side is dorsal in these species. The inversion of the genitalia is, therefore, a diagnostic feature of the species. This is the first documented case of such a species-specific character state in the subfamily Trechinae. An overview of ground beetle groups in which such an inversion of the male genitalia has been documented is provided. Finally, an identification key for all *Trechus* species from Mt. Choke is presented.

## 1. Introduction

The ground beetle genus *Trechus* Clairville, 1806, is of Holarctic origin, however, it is particularly speciose in Ethiopia [1]. The external morphological diversity of this group in Ethiopia is particularly remarkable and has no equivalent in the genus immense distribution area [2,3,4,5]. This enormous morphological diversity has led to the assumption of the presence of several different, earlier diverged Trechini lineages in Ethiopia [3,6,7]. However, based on a molecular phylogenetic study, Faille et al. [8] could show that morphologically highly divergent *Trechus* species, sympatrically occurring in northern and southern Ethiopia, respectively, belong to three geographically separated lineages of *Trechus sensu lato*: the subgenera *Abyssinotus* Quéinnec and Ollivier, 2021 and *Abunetrechus* Schmidt and Faille, 2023 in northern Ethiopia, and the subgenus *Minitrechus* Magrini, Quéinnec and Vigna Taglianti, 2009 in southern Ethiopia. *Abyssinotus* (37 species) and *Minitrechus* (41 species) proved to be particularly diverse in species and morphological forms and together comprise 84% of all *Trechus* species known to date from Ethiopia. In both these subgenera, traits unusual for *Trechus* were developed independently of each other, e.g., the reduction in the number of setae on the elytral disc, the increase in the number of setae in the umbilical series, the reduction in the number of broadened protarsomeres in males, or the extension of the dorsal opening to the phallobase [2,3,6,7,9,10,11,12]. However, previous interpretations of trait evolution in these groups are not based on explicit phylogenetic inference but rather interpretations of the phylogenetic distribution of traits from taxonomic studies.

In the present paper, we point to another unusual character state for trechine beetles, which is developed in two terminal lineages of the subgenus *Abyssinotus* occurring in the Afroalpine zone on Mt. Choke in northern Ethiopia. In species of these lineages, the aedeagus lies with the right side superior in repose and, therefore, opposite to the normal position in Carabidae [13,14]. So far, species with morphologically inverse male genitalia are unknown in the subfamily Trechinae. In addition, based on the newly collected material on Mt. Choke, eight *Trechus* species and three subspecies are newly described, and a key to the species occurring on this volcanic mountain is presented.

## 2. Materials and Methods

This study is based on about 1800 specimens (spms) of the revised and newly described taxa. The specimens are deposited in the following collections:

CAF: Arnaud Faille working collection, Stuttgart, Germany.

CSCHM: Joachim Schmidt working collection, later to be deposited in ZSM.

NHMAA: Natural History Museum, Addis Ababa University.

ZSM: Bavarian State Collection for Zoology, Munich.

Genitalia were prepared after soaking specimens in warm water and mild detergent for one day followed by dissection. Aedeagus and female reproductive tract were cleared in lactic acid for 2–4 days and then washed in 70 percent ethanol. Preparations of the female reproductive tract were then stained for ten minutes with fuchsine solution. After examination, genitalic preparations were stored in Euparal on acetate labels or cards and pinned beneath the specimen from which they had been removed.

Specimens were examined by stereomicroscope Leica M205-C (Leica Microsystems, Wetzlar, Germany). The photographs were taken with a Leica DFC450 digital camera using a motorised focusing drive, light base Leica TL5000 Ergo, diffused light with Leica hood LED5000 HDI, subsequently processed with Leica LAS 4.13 application software, and enhanced with CorelDRAW Graphics Suite X5.

Body size was measured from the tip of the mandibles in open position to the apex of the longer elytron. The width of the head (HW) was measured at its widest part, including the compound eyes. The width of the pronotum (PW) and the width of the elytra (EW) were measured at their widest point. Pronotal length (PL) was measured along the midline. The widths of the apical (PAW) and basal (PBW) margins of the pronotum were measured between the tips of the apical and basal angles, respectively. Elytral length (EL) was measured from the tip of the scutellar shield to the apex of the longer elytron. The length of the aedeagal median lobe (AL) was measured over the greatest distance without taking into account the sagittal aileron.

Terminology of the male and female genitalia and for the interpretation of genitalic orientation follows Jeannel [13], Deuve [14], and Liebherr and Will [15].

## 3. Results

### 3.1. The Trechus lobeliae Subgroup

**Figure 1:**
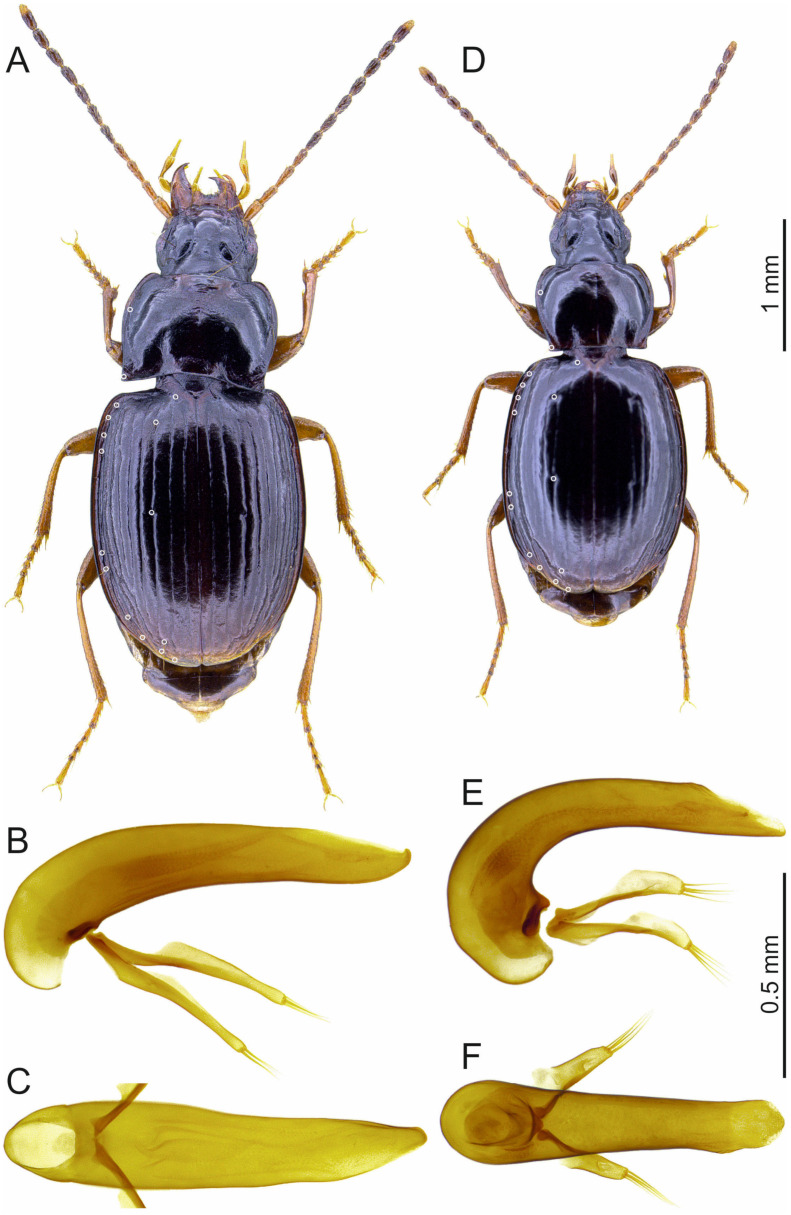
*Trechus* subgenus *Abyssinotus* (*lobeliae* subgroup) male specimens, dorsal aspect of body (**A**,**D**), aedeagus in lateral view (**B**,**E**) and dorsal view (**C**,**F**). (**A**–**C**) *T. lobeliae*, specimen from NE slope of Mt. Choke. (**D**–**F**) *T. inermus* sp. n., paratype from NE slope of Mt. Choke. The white circles point to the insertions of the pronotal lateral setae, parascutellar seta, elytral discal setae, and setae of the umbilicate series.

Species of *Trechus* subgenus *Abyssinotus* sensu Faille et al. [8] are characterised by small to moderate body size, markedly pigmented body, moderately small eyes, transverse pronotum with markedly large laterobasal angles, rectangular to sharp, short elytra with broad shoulders, very short or absent parascutellar stria, anterior two elytral discal setae adjoined to the third stria, second discal seta situated in or before the middle of the elytra, prolegs with one dilated tarsomere in males, aedeagus right side superior in repose, without sagittal aileron, and endophallus completely membranous, without copulatory piece and sclerotized scales (Figure 1). Species of the *T. lobeliae* subgroup share with some species of the *T. basilewskianus* subgroup the upturned position of the aedeagal median lobe (see below) but differ from these and all other *Trechus* s. l. from northern Ethiopia in having a fully membranous endophallus.

This subgroup includes two species endemic to Mt. Choke: *T. lobeliae* and *T. inermus* Schmidt and Faille, sp. n.

#### 3.1.1. *Trechus (Abyssinotus) lobeliae* Quéinnec and Ollivier, 2021

Figure 1A–C.

Citations: *Deuveopsis lobeliae* Quéinnec and Ollivier, 2021 [3], p. 30; locus typicus: Mt. Choke, 3950 m, 10°41′54″ N, 037°50′21″ E.

*Deuveopsis lobeliae* Quéinnec and Ollivier: Merene et al. [1], p. 47.

*Trechus* (*Abyssinotus*) *lobeliae* (Quéinnec and Ollivier): Faille et al. [8], p. 330.

*Trechus* (*Abyssinotus*) *lobeliae* (Quéinnec and Ollivier): Schmidt and Merene [12], p. 353.

Type material: Not studied. Identification is based on the original description, including habitus and male genital figures of the holotype specimen [3] as well as a large series of specimens collected at the type locality and many other locations on Mt. Choke (see Additional material section).

Additional material: 4 males, 1 female, Ethiopia, Amhara, Mt. Choke, western crater valley, alt. 3700–3800 m, 10°41′14″ N 37°50′07″ E, 24.II.2019, leg. D. Hauth, J. Schmidt, Yeshitla M., Yitbarek, W. (CSCHM). A total of 170 spms, W slope Mt. Choke, alt. 3700–3900 m, 10°42′17″ N 37°50′29″ E, 25.II.2019, leg. D. Hauth, J. Schmidt, Yeshitla M., Yitbarek, W. (CAF, CSCHM, NHMAA, ZSM). A total of 312 spms, N slope Mt. Choke, alt. 3800–3950 m, 10°43′16″ N 37°51′15″ E, 26.II.2019, leg. D. Hauth, J. Schmidt, Yeshitla M., Yitbarek, W. (CAF, CSCHM, NHMAA, ZSM). A total of 83 spms, Mt. Choke, north western crater valley, alt. 3780–3900 m, 10°42′12″ N 37°50′58″ E, 27.II.2019, leg. D. Hauth, J. Schmidt, Yeshitla M., Yitbarek, W. (CAF, CSCHM, ZSM). A total of 3 males, 1 female, SW slope Mt. Choke, alt. 3630–3730 m, 10°38′02″ N 37°50′08″ E, 28.II.2022, leg. D. Hauth, J. Schmidt, Yeshitla M., Yitbarek, W. (CSCHM). A total of 15 spms, NE slope Mt. Choke, above Felege Birhan, alt. 3750–3850 m, 10°42′13″ N 37°56′32″ E, 30.IV.2022, leg. J. Schmidt, Yeshitla M. (CSCHM). A total of 1 male, Ethiopia, Amhara, E slope Mt. Choke, Wondasha Guskuam, alt. 3650 m, 10°41′05″ N 37°59′21″ E, 2.V.2022, leg. J. Schmidt, Yeshitla, M. (CSCHM). A total of 101 spms, NE slope Mt. Choke, alt. 3700–3880 m, 10°42′56″ N 37°55′16″ E, 4.V.2022, leg. J. Schmidt, Yeshitla M. (CSCHM, ZSM). A total of 81 spms, NE slope Mt. Choke, above Gumadur [Shotele], alt. 3750–3850 m, 10°44′10″ N 37°53′48″ E, 5.V.2022, leg. J. Schmidt, Yeshitla M. (CSCHM, ZSM). A total of 6 males, 2 females, Mt. Choke, north eastern crater valley, alt. 3700–3800 m, 10°42′59″ N 37°54′13″ E, 6.V.2022, leg. J. Schmidt, Yeshitla M. (CSCHM). A total of 51 spms, W slope Mt. Choke, “Shoa Kidaneberet” [Kidanemehret] valley, alt. 3700–3800 m, 10°39′08″ N 37°49′45″ E, 8.V.2022, leg. J. Schmidt, Yeshitla M. (CSCHM, ZSM). A total of 126 spms, N slope Mt. Choke, alt. 3750–3850 m, 10°43′51″ N 37°52′15″ E, 9.V.2022, leg. J. Schmidt, Yeshitla M. (ZSM). A total of 2 males, W slope Mt. Choke, alt. 3680–3780 m, 10°40′11″ N 37°48′33″ E, 11.V.2022, leg. J. Schmidt, Yeshitla M. (CSCHM, ZSM).

Description. See Quéinnec et al. [3]: 30–32.

Identification: See description of *T. inermus* sp. n. and key to species, below.

**Figure 2:**
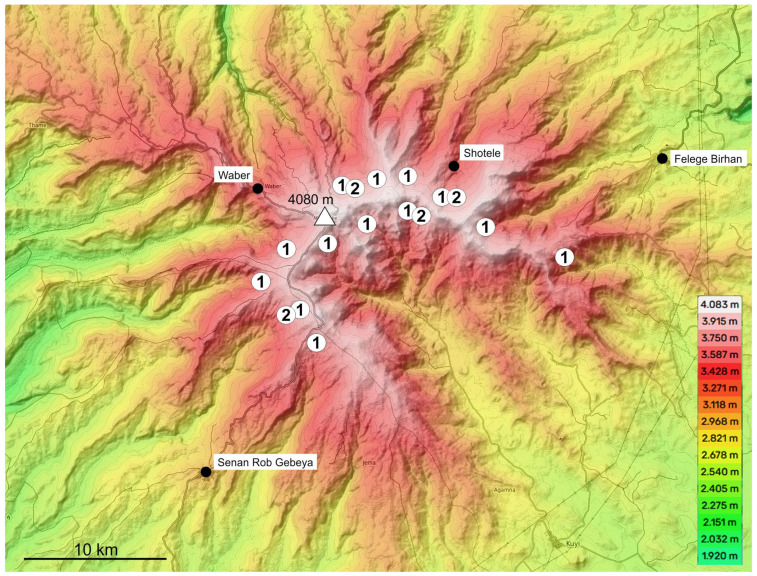
Topographic map of Mt. Choke (highest point marked by a triangle), showing sampling localities of the species of the *T. lobeliae* subgroup of *Abyssinotus* (white circles). **1**, *T. lobeliae*. **2**, *T. inermus* sp. n. The black circles mark locations of important settlements on slope of Mt. Choke. Base map from Topographic-map.com (accessed on 1 January 2024).

Distribution: Widespread along the crater rim of Mt. Choke (Figure 2).

**Figure 3:**
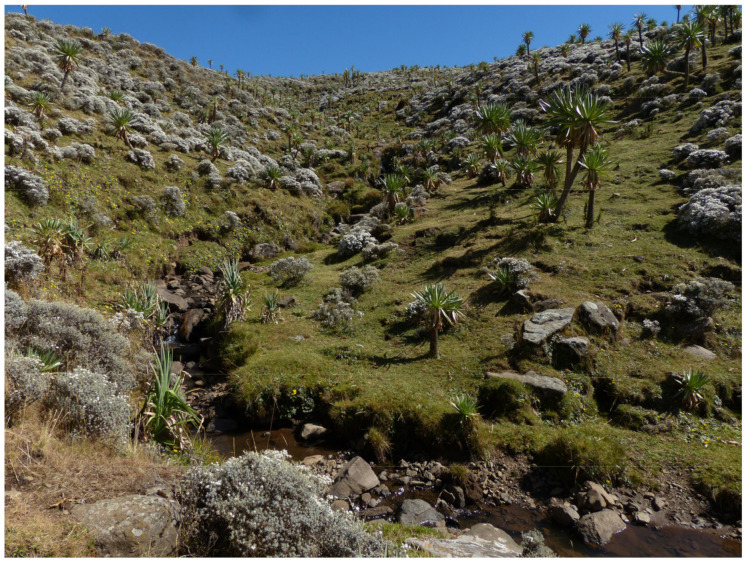
North-exposed slope of Mt. Choke at elevations 3700–3900 m, with sampling sites of species from the *lobeliae* and *basilewskianus* subgroups of *Abyssinotus* (February 2019). *Trechus lobeliae* and *T. inermus* sp. n. were sifted from the humus-rich soil beneath *Helichrysum* shrubs and giant lobelia, and on shaded bank slopes above the stream water. *Trechus adsuetus* sp. n. and *T. sebsebei extremus* ssp. n. were found on steep stream banks, and here exclusively in shady places under stones and in the wet soil close to the water.

Habitat: Based on our own field studies in the Afroalpine and Afromontane zones of Mt. Choke, at altitudes between 3600 and 4000 m, *T. lobeliae* is a very common, mesophilous species. It is shifted in large numbers from raw humus in shady places under *Erica* trees and various shrubs and perennials (Figure 3).

#### 3.1.2. *Trechus (Abyssinotus) inermus* Schmidt and Faille, sp. n.

Figure 1D–F.

Type material: Holotype male, with label data: Ethiopia, Amhara, NE slope Mt. Choke, alt. 3700–3880 m, 10°42′56″ N 37°55′16″ E, 4.V.2022, leg. J. Schmidt, Yeshitla M. (CSCHM).

Paratypes: 54 males, 39 females, same data as holotype (CAF, CSCHM, NHMAA, ZSM). A total of 1 male, 1 female, N slope Mt. Choke, alt. 3800–3950 m, 10°43′16″ N 37°51′15″ E, 26.II.2019, leg. D. Hauth, J. Schmidt, Yeshitla M., Yitbarek, W. (CSCHM). A total of 1 male, Mt. Choke, north eastern crater valley, alt. 3700–3800 m, 10°42′59″ N 37°54′13″ E, 6.V.2022, leg. J. Schmidt, Yeshitla M. (CSCHM). A total of 3 males, 2 females, W slope Mt. Choke, “Shoa Kidaneberet” [Kidanemehret] valley, alt. 3700–3800 m, 10°39′08″ N 37°49′45″ E, 8.V.2022, leg. J. Schmidt, Yeshitla M. (CSCHM).

Etymology: The specific epithet is derived from the Latin adjective “inermus” (unarmed) and refers to the reduced copulatory piece of the male genitalia.

Description: Body length: 2.8–3.4 mm.

Proportions (n = 10): PW/HW = 1.37–1.46 (Ø = 1.40); PW/PL = 1.34–1.43 (Ø = 1.37); PW/PBW = 1.20–1.27 (Ø = 1.22); PBW/PAW = 1.16–1.21 (Ø = 1.19); EW/PW = 1.49–1.57 (Ø = 1.52); EL/EW = 1.21–1.26 (Ø = 1.24); EL/AL = 1.98–2.14 (Ø = 2.05).

Colour: Blackish brown; mandibles, basal and apical maxillary palpomeres, scape and basal portions of antennomeres 2 and 3, and legs lighter brown.

Microsculpture: Head with moderately deep engraved, isodiametric sculpticells on the neck and with shallower, slightly transverse sculpticells on the disc, supraorbital area and clypeus. Pronotum with very small, finely engraved, somewhat irregularly shaped sculpticells on the disc and more deeply engraved sculpticells in the pronotal basolateral foveae and near the basal margin. Elytral intervals with finely engraved, slightly transverse sculpticells; sculpticells in females larger than in males.

Head: Medium size for *Trechus* (s. l.). Right mandible tridentate, very similar to that of *T. abyssinicus* Quéinnec and Ollivier and *T. niloticus* Quéinnec and Ollivier (see [3], p. 12) with a prominent and sharp premolar, and a bifid retinaculum. Labrum with moderately emarginate apical margin, with six setae near apical margin. Clypeus with two setae on each side. Eyes moderately small, about 1.5 times as long as tempora, convex and slightly protruding. Two supraorbital setae on each side, in normal position for Trechina. Supraorbital furrows moderately deep, +/− evenly bent on disc. Middle of head and supraorbital area moderately convex and elevated. Tempora moderately convex, distinctly wrinkled up to the neck, with distinct micro-setation. Antennae moderately short, with first, second, and third antennomeres almost the same length.

Prothorax: Pronotum moderately large, transverse, without pilosity, with base distinctly broader than apical margin, broadest before middle. Disc moderately convex and elevated. Anterior margin slightly or moderately concave, with moderately protruding anterior angles. Basal margin straight in the middle, with outer quarters more or less distinctly shifted posteriad. Lateral margin almost convex in anterior 2/3, concave before laterobasal angles; the tip of the latter slightly protruding laterally. Laterobasal angles large, rectangular, or slightly acute. Marginal gutter narrow in anterior 4/5, widened at laterobasal angles. Median longitudinal impression very finely incised on disc, disappearing near apex, somewhat deeper before base. Anterior transverse impression absent; posterior one very shallow, indistinct, smooth. Laterobasal foveae large, moderately deep, smooth, delimited from the lateral gutter by a wide, moderately elevated area. Lateral and laterobasal setae present, the former at the maximum width of the pronotum. Proepisternum glabrous and smooth.

Pterothorax: Elytra glabrous, with dorsal surface moderately elevated, in dorsal view short oval, broadest in the middle, shoulders broad and shortly rounded, apical sinuation distinct, apex rounded with indication of a very obtuse apical angle. Striae 1–8 complete, striae 1–3, and 8 moderately deep impressed, 4–7 finer, 5–7 indistinct in some specimens, finely punctate, intervals slightly convex, parascutellar stria absent. Clearly deep recurrent preapical stria connected to the fifth or seventh stria, or isolated. Parascutellar seta present. Discal setae located in the third interval, attached to the third stria; anterior seta located near the end of the anterior elytral 1/6 or 1/7; second seta located in the middle of elytra; posterior discal seta (=subapical seta near the end of third stria) present, located about 1/8 the length of the elytra from the elytral apex; subapical seta of the recurrent stria isolated, distant from this stria by about one diameter of the setiferous pore. Number and position of setae of marginal umbilicate series as in *Trechus* s. str. Metepisternum very short, glabrous and smooth, with outer margin about as long as anterior margin.

Legs: Short and robust. Protibia dilated towards apex, almost straight on external margin, with a developed longitudinal groove on dorsal surface, with a few very fine setae on anterior surface near apex. One basal protarsomere of males dilated and dentate at inner apical margin.

Aedeagus: Length 0.80–0.85 mm. Median lobe comparatively large and robust, in lateral view markedly bent in basal half, almost straight towards apex, without distinct terminal lamella; in dorsal view broad at base, moderately slender in middle and front, on left side with a slight convexity before apex, the latter short and blunt. Basal bulb of median lobe rather large, without sagittal aileron. Endophallus without copulatory piece, densely covered with small, sclerotized scales. Parameres each with four apical setae.

Differential diagnosis: Because of the absence of an endophallic copulatory piece, the short elytra, and the largely developed pronotal laterobasal angles, the new species is very similar to *T. lobeliae*. It is easily distinguished from this species by a shorter body length (2.8–3.4 mm, instead of 3.6–4.2 mm in *T. lobeliae*), darker antennae, a smaller pronotum with less large and acute laterobasal angles, elytra with smaller shoulders, less deeply engraved striae and anterior two discal setae that are not shifted anteriad (the second seta situated in the middle of elytra, and not distinctly before the middle as in *T. lobeliae*), and the aedeagus larger relative to body length, with the median lobe much more markedly bent (lateral view) and without a distinct apical lamella. *Trechus inermus* sp. n. can be distinguished from species in the *T. basilewskianus* subgroup by the presence of large laterobasal angles and the absence of an endophallic copulatory piece, and from all other *Trechus* species from Mt. Choke not belonging to the *T. lobeliae* and *T. basilewskianus* subgroups by the aedeagus with median lobe right side superior in repose.

Distribution: The new species was found in the upper parts of several valleys along the western and norther slopes of Mt. Choke, as well as on the north eastern side of the crater (Figure 2).

Habitat: Specimens of the new species were collected with those of *T. lobeliae* (Figure 3 and see above, habitat description of *T. lobeliae*). According to current knowledge, this is a mesophilic species, adapted to life in humus-rich soil and shady locations.

### 3.2. The Trechus basilewskianus Subgroup (Abayopsis Quéinnec and Ollivier, 2021)

Species of *Trechus* subgenus *Abyssinotus* sensu Faille et al. [8], characterised by a moderate size, a well pigmented body, rather small and flat eyes, an *Agonum*-like discoid pronotum with clearly obtuse or rounded laterobasal angles, slender elytra, an absent parascutellar stria, anterior two elytral discal setae situated at the level of the fourth interval and connecting the third with the fourth stria, the first discal seta situated at the end of the anterior elytral 1/9, the second discal seta situated distinctly before the middle of elytra, prolegs with two dilated tarsomeres in males, aedeagus either with the left or right side superior in repose, median lobe with a very small or absent sagittal aileron and with a long apical lamella clearly bent upwards, endophallus largely sclerotized, with a +/− long copulatory piece.

This subgroup comprises eight species and three additional subspecies endemic to Mt. Choke. All species are very similar to each other in terms of external characters but the morphology of the male genitalia differs markedly. In six of the species and their respective subspecies, the aedeagus is developed with the right side superior in repose: *T. b. basilewskianus* Geginat, 2008, *T. b. extendipenis* Schmidt and Faille ssp. n., *T. diversus* Schmidt and Faille sp. n., *T. infrequens* Schmidt and Faille sp. n., *T. inversus* Schmidt and Faille sp. n., *T. sebsebei* Schmidt and Faille sp. n., *T. sebsebei curvipenis* Schmidt and Faille ssp. n., *T. sebsebei extremus* Schmidt and Faille ssp. n., and *T. waberense* Schmidt and Faille sp. n. In two of the species, the aedeagus is developed with the left side superior in repose (=normal position in Trechinae): *T. adsuetus* Schmidt and Faille sp. n. and *T. sinuatipenis* Schmidt and Faille sp. n.

#### 3.2.1. *Trechus (Abyssinotus) basilewskianus* Geginat, 2008

**Figure 4:**
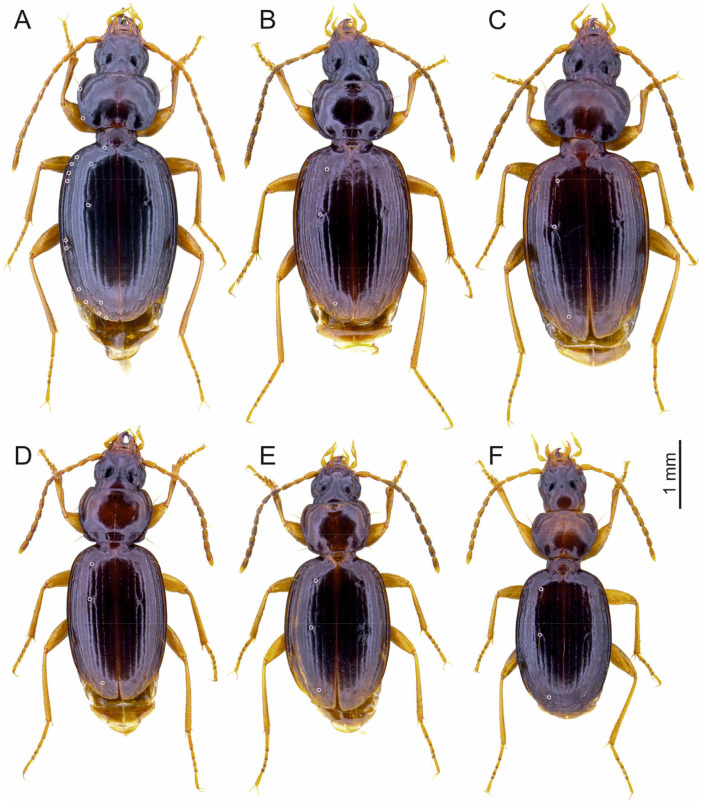
*Trechus* subgenus *Abyssinotus* (*basilewskianus* subgroup) male specimens, dorsal aspect of the body. (**A**) *T. basilewskianus*, specimen from E slope of Mt. Choke, above Felege Birhan. (**B**) *T. sebsebei* sp. n., paratype from western crater valley of Mt. Choke. (**C**) *T. waberense* sp. n., holotype. (**D**) *T. inversus* sp. n., paratype. (**E**) *T. infrequens* sp. n., paratype. (**F**) *T. diversus* sp. n., paratype. White circles indicate insertions of pronotal lateral setae, parascutellar seta, elytral discal setae, and setae of the umbilicate series (in Figures (**B**–**F**) only insertions of elytral discal setae are shown).

**Figure 5:**
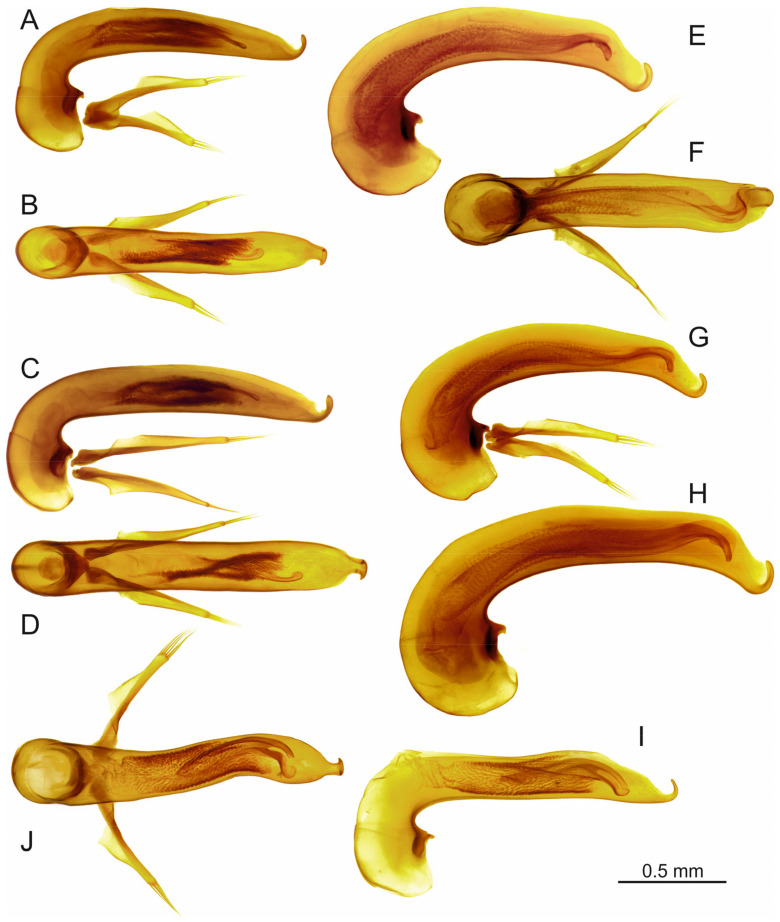
*Trechus* subgenus *Abyssinotus* (*basilewskianus* subgroup), aedeagus, lateral aspect (**A**,**C**,**E**,**G**–**I**) and dorsal aspect (**B**,**D**,**F**,**J**). (**A**,**B:** *T. b. basilewskianus*, specimen from E slope of Mt. Choke, above Felege Birhan. (**C**,**D**) *T. b. extendipenis* ssp. n., paratype. (**E**,**F**) *T. sebsebei* sp. n., paratype from western crater valley of Mt. Choke. (**G**) *T. sebsebei curvipenis* ssp. n., paratype. (**H**) *T. sebsebei extremus* ssp. n., paratype. (**I**,**J**) *T. waberense* sp. n., holotype.

Figure 4A and Figure 5A,B.

Citations: *Cothresia minuta* Basilewsky, 1974 [16], p. 151; locus typicus: Mts Choché, 3500–4000 m, “10°44′ N 37°55′ E” (see section remarks to the type locality, below).

*Trechus basilewskianus* Geginat, 2008 [17], p. 124 [replacement name].

*Deuveopsis* (*Abayopsis*) *basilewskianus* (Geginat): Quéinnec et al. [3], p. 33, partim. Remarks: In addition to the type specimens, Quéinnec et al. [4] recorded several “*D. basilewskianus*” specimens from their sampling points “station I”, “IV”, and “VI”, which are located on the western rim of the Choke crater. This location is not part of the range of *T. basilewskianus*. The specimen cited most probably belongs to *T. sebsebei* sp. n. (see below).

*Deuveopsis* (*Abayopsis*) *minuta* (Basilewsky): Merene et al. [1], p. 47, partim. Remarks: These authors refer to the same material as Quéinnec et al. [3]; see comments above.

*Trechus* (*Abyssinotus*) *basilewskianus* Geginat: Faille et al. [8], p. 330.

Type material: Not studied. Identification is based on the description and redescription of the species, including the photograph of the male genitalia of the holotype specimen [15,16], as well as additional specimens recently collected at the type locality.

Remarks on the type locality: The type series of *T. basilewskianus* was collected by R.O.S. Clarke during his 1972 expedition to the Amhara region of Ethiopia. According to the original description, he collected the specimens near the summit of Mt. Choke and near the camp site, on 17th of December 1972 [14]. Along with *T. basilewskianus*, Clarke collected other trechine species on the same day, namely *T. dimorphicus* Pawłowski, 2001 and *T. gigas* Pawłowski, 2001 [18]. The latter two species have been identified as local endemics of some valleys on the eastern slope of Mt. Choke [4]. During our own field observations, we collected specimens of *T. basilewskianus* together with *T. dimorphicus* along a valley of Mt. Choke above the town of Felege Birhan (see additional material section). This valley has been identified as the type locality for *T. dimorphicus* [4]. It is, therefore, very likely that this valley is also the type locality for *T. basilewskianus*. However, Basilewsky [16]: 154 recorded the following coordinates for the side of Clarke’s camp: 10°40′ N 37°55′ E. These coordinates refer to a more northerly location on Mt. Choke where *T. dimorphicus* is absent and its geographic vicariant taxon *T. salomon variipenis* Schmidt, 2024 is distributed [4]. We suspect that these geographical deviations were caused by inaccuracies in the recording of geographic position during Clarke’s expedition over 50 years ago. This assumption is further supported by the male genital morphology of the different populations of *T. basilewskianus*. The aedeagal median lobe of the holotype specimen was figured by Basilewsky [16]: 152 and photographed by Geginat [17]: 125. From these figures, the aedeagus of the holotype of *T. basilewskianus* is morphologically identical to specimens collected in the valley above Felege Birhan, but differs significantly from those found in the neighbouring valley to the north (see distribution and geographic variation, below).

Additional material: 2 males, 2 females, Ethiopia, Amhara, NE slope Mt. Choke, above Felege Birhan, alt. 3750–3850 m, 10°42′13″ N 37°56′32″ E, 30.IV.2022, leg. J. Schmidt, Yeshitla M. (CSCHM). A total of 9 males, 12 females, Mt. Choke, north eastern crater valley, alt. 3700–3800 m, 10°42′59″ N 37°54′13″ E, 6.V.2022, leg. J. Schmidt, Yeshitla M. (CSCHM).

Redescription. Body length: 4.2–4.9 mm.

Proportions (n = 10): PW/HW = 1.44–1.51 (Ø = 1.48); PW/PL = 1.35–1.44 (Ø = 1.41); PW/PBW = 1.22–1.30 (Ø = 1.26); PBW/PAW = 1.15–1.27 (Ø = 1.20); EW/PW = 1.40–1.48 (Ø = 1.44); EL/EW = 1.43–1.48 (Ø = 1.46); EL/AL = 1.76–1.83 (Ø = 1.79).

Colour: Dorsal surface dark brown with pronotum reddish brown in some specimens, and with blurred elytral apex lighter than elytral disc; mandibles, labrum, clypeus and distal antennomeres medium brown; palps, 3–4 basal antennomeres and legs yellowish brown.

Microsculpture: Head with large, deeply engraved, almost isodiametric sculpticells on neck, disc, and supraorbital area; sculpticells on clypeus similar in shape but smaller. Pronotum with moderately large, moderately deep, somewhat irregularly shaped sculpticells; sculpticells more deeply engraved in pronotal basolateral foveae. Elytral intervals with moderately deep, small and transverse sculpticells in both sexes.

Head: Size rather small. Right mandible tridentate (see [3], p. 12, with prominent, sharp, premolar, and an indistinctly bifid retinaculum. Labrum with moderately emarginate apical margin, with six setae near apical margin. Clypeus with two setae on each side. Eyes small, about as long as tempora, very slightly convex. Two supraorbital setae on each side in normal position for Trechina. Supraorbital furrows moderately deep, +/− evenly bent on disc. Middle of head and supraorbital area moderately convexly elevated. Tempora moderately convex, distinctly wrinkled up to the neck, with distinct micro-setation. Antennae moderately slender, with the third antennomere about 1.7x longer than the second.

Prothorax: Pronotum moderately large, transversely discoidal, without pilosity, with base somewhat broader than apical margin, broadest before middle. Disc moderately convexly elevated. Anterior margin straight in middle, laterally protruded towards anterior angles, the latter shortly rounded. Basal margin straight or slightly concave in middle, with outer quarters convexly rounded and with laterobasal angle distinctly shifted anterad. Lateral margin convex almost throughout, with small and obtuse laterobasal angles very slightly protruded laterally. Marginal gutter narrow in anterior 2/3, widened before laterobasal angles, joining the laterobasal foveae. Median longitudinal impression finely incised, disappearing near apex and base. Anterior transverse impression very flat, indistinct; posterior one broad but shallow, smooth. Laterobasal foveae large, moderately deep, smooth, broadly connected to lateral gutter. Lateral and laterobasal setae present, the former situated at the maximum width of pronotum. Proepisternum glabrous and smooth.

Pterothorax: Elytra glabrous, with dorsal surface moderately convexly elevated, in dorsal view slender oval, broadest about at middle, shoulders broadly rounded, apical sinuation distinct, apex rectangular or slightly pointed. Striae 1–8 complete, finely impressed and finely punctate, 6 and 7 a little more finely impressed than the internal striae, 8 markedly deeper towards the base; intervals slightly convex, parascutellar stria absent. Recurrent preapical stria markedly deep, connected to the fifth stria. Parascutellar seta present. Anterior two discal setae situated at level of fourth interval and connecting the third with the fourth stria; first discal seta situated at the end of the anterior elytral 1/9, second discal seta situated distinctly before the middle of elytra; posterior discal seta present, located about 1/8 of elytral length from elytral apex; subapical seta of the recurrent stria very fine, isolated, distant from this stria by about one diameter of the setiferous pore. Number and position of setae of marginal umbilicate series as in *Trechus* s. str. Metepisternum very short, glabrous and smooth, with outer margin about as long as anterior margin.

Legs: Moderately slender. Protibia dilated towards apex, almost straight on outer margin, with a developed longitudinal groove on dorsal surface, and with a few fine setae on anterior surface near apex. Two basal protarsomeres of males dilated and dentate at inner apical margin.

Aedeagus: Length 1.35–1.52 mm. Aedeagus with upper right side superior in repose. Median lobe elongated, in lateral view markedly bent in basal half, very slightly bent towards apex, with long terminal lamella curved upwards and slightly inverted; in dorsal view slender throughout, suggestively sinusoidal, the terminal lamella lingulate. Basal bulb of median lobe rather small, without sagittal aileron. Endophallus entirely covered with large, sclerotized scales, with a rod-shaped copulatory piece; the latter ending in a small apical hook (dorsal view). Parameres each with four apical setae.

Identification: See key to species below.

**Figure 6:**
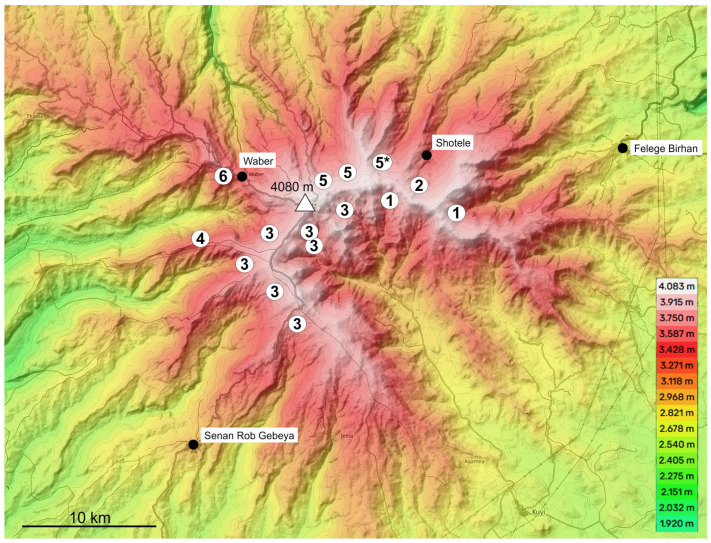
Topographic map of Mt. Choke (highest point marked by a triangle), showing the sampling localities of species of the *T. basilewskianus* subgroup of *Abyssinotus* (white circles). **1**, *T. b. basilewskianus*. **2**, *T. b. extendipenis* ssp. n. **3**, *T. sebsebei* sp. n. (s. str.). **4**, *T. s. curvipenis* ssp. n. **5**, *T. s. extremus* ssp. n. 5*, population with male genital shape intermediate to *T. sebsebei* sp. n. (s. str.) and *T. s. extremus* ssp. n. (see text for details). **6**, *T. waberense* sp. n. The black circles mark locations of important settlements on slope of Mt. Choke. Base map from Topographic-map.com.

Distribution and geographic variation: *Trechus basilewskianus* was found in the higher parts of three valleys along the eastern rim of the crater of Mt. Choke (Figure 6). Specimens from populations found in the valley above Felege Birhan (=putative type locality, see remarks above) and across the opposite side of the eastern crater rim share the same male genital morphology and are, therefore, considered to be representatives of *T. basilewskianus* sensu stricto. Specimens from a population found in a valley of Mt. Choke further north are characterised by a slightly different male genital morphology and are, therefore, described as members of a distinct subspecies, below.

Habitat: During our fieldwork, we found *T. basilewskianus* in coarse gravel and loose, moist soil on the banks of shaded sections of small streams at altitudes of 3700–3850 m. On the basis of these observations, *T. basilewskianus* is considered to be a strictly hygrophilous species.

#### 3.2.2. *Trechus (Abyssinotus) basilewskianus extendipenis* Schmidt and Faille, ssp. n.

Figure 5C,D.

Type material: Holotype male, with label data: Ethiopia, Amhara, NE slope Mt. Choke, alt. 3700–3880 m, 10°42′56″ N 37°55′16″ E, 4.V.2022, leg. J. Schmidt, Yeshitla M. (CSCHM).

Paratypes: 4 males, 5 females, same data as holotype (CSCHM).

Etymology: The specific epithet refers to the extended median lobe of the aedeagus compared to the nominotypical subspecies.

Description. Body length: 4.3–4.6 mm.

Proportions (n = 10): PW/HW = 1.40–1.47 (Ø = 1.44); PW/PL = 1.32–1.40 (Ø = 1.36); PW/PBW = 1.18–1.26 (Ø = 1.22); PBW/PAW = 1.19–1.24 (Ø = 1.21); EW/PW = 1.41–1.46 (Ø = 1.44); EL/EW = 1.49–1.51 (Ø = 1.50); EL/AL = 1.62–1.67 (Ø = 1.65).

Colour and microsculpture: As described for *T. basilewskianus* s. str.

Head and pronotum: As described for *T. basilewskianus* s. str.

Pterothorax: Elytra, on average, slightly more elongate. All other characters as described for *T. basilewskianus* s. str.

Legs: As described for *T. basilewskianus* s. str.

Aedeagus: Length 1.50–1.65 mm. Aedeagus with right side superior in repose. Median lobe elongated, in lateral view markedly bent in basal third, almost straight towards apex, with long terminal lamella, curved upwards and slightly inverted; in dorsal view straight and moderately slender, the terminal lamella lingulate. Basal bulb of median lobe small, without sagittal aileron. Endophallus and parameres as described for *T. basilewskianus* s. str.

Identification: Differs from the nominotypical form mainly in the aedeagal median lobe, which is longer relative to body length (EL/AL < 1.7 instead of > 1.7 in *T. basilewskianus* s. str.), slightly more robust, and more straight in lateral and dorsal view. The elytra are more slender on average (Ø EL/EW = 1.50 instead of 1.46 in *T. basilewskianus* s. str.).

Distribution: Probably endemic to a single valley system on the northeastern slope of Mt. Choke (Figure 6).

Habitat: As for the nominotypical subspecies.

#### 3.2.3. *Trechus (Abyssinotus) sebsebei* Schmidt and Faille, sp. n.

Figure 4B and Figure 5E,F.

Type material: Holotype male, with label data: Ethiopia, Amhara, Mt. Choke, crater valley, alt. 3780–3900 m, 10°42′12″ N 37°50′58″ E, 27.II.2019, leg. D. Hauth, J. Schmidt, Yeshitla M., Yitbarek, W. (CSCHM).

Paratypes: 35 males, 31 females, same data as holotype (CAF, CSCHM, NHMAA, ZSM). A total of 8 males, 3 females, Mt. Choke, crater valley, alt. 3700–3800 m, 10°41′14″ N 37°50′07″ E, 24.II.2019, leg. D. Hauth, J. Schmidt, Yeshitla M., Yitbarek, W. (CSCHM). A total of 25 males, 17 females, W slope Mt. Choke, alt. 3700–3900 m, 10°42′17″ N 37°50′29″ E, 25.II.2019, leg. D. Hauth, J. Schmidt, Yeshitla M., Yitbarek, W. (CSCHM, NHMAA, ZSM). A total of 9 males, 13 females, SW slope Mt. Choke, alt. 3630–3730 m, 10°38′02″ N 37°50′08″ E, 28.II.2022, leg. D. Hauth, J. Schmidt, Yeshitla M., Yitbarek, W. (CSCHM). A total of 1 male, 1 female, Mt. Choke, western crater valley, alt. 3500–3600 m, 10°41′00″ N 37°50′35″ E, 01.V.2022, leg. J. Schmidt, Yeshitla M., (CSCHM). A total of 9 males, 6 females, W slope Mt. Choke, alt. 3680–3780 m, 10°40′11″ N 37°48′33″ E, 11.V.2022, leg. J. Schmidt, Yeshitla M. (CSCHM). A total of 23 males, 18 females, W slope Mt. Choke, “Shoa Kidaneberet” valley, alt. 3700–3800 m, 10°39′08″ N 37°49′45″ E, 8.V.2022, leg. J. Schmidt, Yeshitla M. (CAF, CSCHM, NHMAA, ZSM).

Etymology: The new species is dedicated to the outstanding botanist Sebsebe Demissew, Professor of Plant Systematics and Biodiversity at Addis Ababa University.

Description: Body length: 4.3–5.1 mm.

Proportions (n = 10): PW/HW = 1.46–1.51 (Ø = 1.48); PW/PL = 1.36–1.44 (Ø = 1.41); PW/PBW = 1.19–1.24 (Ø = 1.22); PBW/PAW = 1.22–1.27 (Ø = 1.24); EW/PW = 1.43–1.49 (Ø = 1.45); EL/EW = 1.40–1.48 (Ø = 1.44); EL/AL = 1.70–1.78 (Ø = 1.74).

Colour and microsculpture: As described for *T. basilewskianus*.

Head and prothorax: As described for *T. basilewskianus*.

Pterothorax: Elytra slender oval, with an obtuse or shortly rounded sutural angle. All other characters as described for *T. basilewskianus*.

Legs: As described for *T. basilewskianus*.

Aedeagus: Length 1.45–1.60 mm. Aedeagus with right side superior in repose. Median lobe large, moderately robust and elongated, in lateral view 180°curved in basal half, moderately bent towards apex; long apical lamella strongly bent upwards and backwards, resulting in a large apical hook. Median lobe tubular in dorsal view, the sides straight and almost parallel, the terminal lamella lingulate. Basal bulb of median lobe moderately large, without sagittal aileron. Endophallus very completely covered with sclerotized scales, extending from the basal bulb towards the base of the terminal lamella, and with a very long, wide, flat ribbon-shaped copulatory piece, which ends in a large hook; this hook-shaped end of the copulatory piece has the same shape as the apical lamella of the median lobe (lateral view), but is bent in the opposite direction to it. Parameres slender, each with four apical setae.

Differential diagnosis: The new species is externally very similar to *T. basilewskianus*, but on average larger, with a more rounded elytral apex. It can easily be distinguished by the male genitalia, which is larger and more markedly bent, with a larger basal bulb and a larger terminal lamella; the latter is more markedly curved (lateral view). The endophallic copulatory piece of *T. sebsebei* sp. n. is broad and flat ribbon-shaped instead of narrow and rod-shaped, much larger, and characterised by a much larger terminal hook. For differentiation from other members of the *basilewskianus* subgroup, see the description of these species and the key to species below.

Distribution and geographical variation: Widely distributed in the valleys along the western parts of the crater of Mt. Choke (Figure 6). A population characterised by a smaller penis was found on a mountain range extending from the Choke crater further west. On the northern slope of Mt. Choke, on the other hand, populations with a larger penis have been found. The two forms are described below as independent subspecies.

Habitat: As described for *T. basilewskianus*.

#### 3.2.4. *Trechus (Abyssinotus) sebsebei curvipenis* Schmidt and Faille, ssp. n.

Figure 5G.

Type material: Holotype male, with label data: Ethiopia, Amhara, W slope Mt. Choke, alt. 3370 m, 10°38′07″ N 37°45′51″ E, 23.II.2019, leg. D. Hauth, J. Schmidt, Yeshitla M., Yitbarek, W. (CSCHM).

Paratypes: 29 males, 25 females, same data as holotype (CSCHM, NHMAA, ZSM).

Etymology: The specific epithet refers to the markedly bent median lobe of the aedeagus.

Description. Body length: 3.9–4.5 mm.

Proportions (n = 10): PW/HW = 1.44–1.48 (Ø = 1.46); PW/PL = 1.41–1.44 (Ø = 1.42); PW/PBW = 1.20–1.24 (Ø = 1.23); PBW/PAW = 1.22–1.25 (Ø = 1.23); EW/PW = 1.45–1.53 (Ø = 1.49); EL/EW = 1.38–1.41 (Ø = 1.39); EL/AL = 1.74–1.79 (Ø = 1.76).

Colour and microsculpture: As described for the nominotypical form.

Head and pronotum: As described for the nominotypical form.

Pterothorax: Elytra normally oval, not particularly elongate. All other characters as described for the nominotypical form.

Legs: As described for the nominotypical form.

Aedeagus: Length 1.32–1.42 mm. General shape of median lobe, endophallus, and parameres as described for the nominotypic form.

Identification: Differs from the nominotypic form by having a smaller body on average (3.9–4.5 mm instead of 4.3–5.1 mm in *T. sebsebei* s. str.), a shorter aedeagal median lobe (1.32–1.42 mm instead of 1.45–1.60 mm in *T. sebsebei* s. str.) and shorter elytra (Ø EL/EW = 1.39 instead of 1.44 in *T. basilewskianus* s. str.).

Distribution: Probably endemic to a single mountain range extending from the Choke crater further west (Figure 6).

Habitat: As in the nominotypical subspecies.

#### 3.2.5. *Trechus (Abyssinotus) sebsebei extremus* Schmidt and Faille, ssp. n.

Figure 5H.

Type material: Holotype male, with label data: Ethiopia, Amhara, N slope Mt. Choke, alt. 3750–3850 m, 10°43′51″ N 37°52′15″ E, 9.V.2022, leg. J. Schmidt, Yeshitla M. (CSCHM).

Paratypes: 16 males, 8 females, same data as holotype (CSCHM). A total of 2 males, 2 females, N slope Mt. Choke, alt. 3800–3950 m, 10°43′16″ N 37°51′15″ E, 26.II.2019, leg. D. Hauth, J. Schmidt, Yeshitla M., Yitbarek, W. (CSCHM).

Additional material: 13 males, 10 females, NE slope Mt. Choke, above Gumadur [Shotele], alt. 3750–3850 m, 10°44′10″ N 37°53′48″ E, 5.V.2022, leg. J. Schmidt, Yeshitla M. (CSCHM).

Etymology: The specific epithet refers to the markedly large median lobe of the aedeagus.

Description. Body length: 4.5–5.0 mm.

Proportions (n = 10): PW/HW = 1.42–1.48 (Ø = 1.46); PW/PL = 1.41–1.45 (Ø = 1.43); PW/PBW = 1.22–1.26 (Ø = 1.24); PBW/PAW = 1.21–1.26 (Ø = 1.23); EW/PW = 1.47–1.52 (Ø = 1.49); EL/EW = 1.40–1.50 (Ø = 1.46); EL/AL = 1.51–1.61 (Ø = 1.59).

Colour and microsculpture: As described for the nominotypic form.

Head, pronotum, elytra, and legs: As described for the nominotypic form.

Aedeagus: Length 1.75–1.85 mm. General shape of median lobe, endophallus and parameres as described for the nominotypic form.

Identification: Differs from the other subspecies of *T. sebsebei* s. l. by the larger aedeagal median lobe (length > 1.7 mm instead of 1.45–1.60 mm in *T. sebsebei* s. str. and 1.32–1.42 mm in *T. s. curvipenis* ssp. n.), and from *T. s. curvipenis* ssp. n. additionally by a larger body on average (4.5–5.0 mm instead of 3.9–4.5 mm in *T. sebsebei* ssp. n.) and longer elytra (Ø EL/EW = 1.46 instead of 1.39 in *T. curvipenis* ssp. n.).

Distribution and geographical variation: Endemic to the northern slope of Mt. Choke (Figure 6). In the valley above the village of Gumadur (Shotele), a population was found in which the length of the median lobe of the aedeagus is 1.60–1.75 mm and, thus, falls between that of *T. sebsebei* s. str. and *T. extremus* ssp. n. These specimens are not included in the type series of the new subspecies (see additional material section). Interestingly, the range of the population with intermediate male genital characters is not geographically located between the ranges of the two subspecies but is at the north eastern limit of the range of *T. sebsebei* s. l. (Figure 6).

Habitat: As for the nominotypical subspecies (photo of the habitat see Figure 3).

#### 3.2.6. *Trechus (Abyssinotus) waberense* Schmidt and Faille, sp. n.

Figure 4C and Figure 5J,I.

Type material: Holotype male, with label data: Ethiopia, Amhara, N slope Mt. Choke, N of Waber, alt. 3450–3600 m, 10°44′48″ N 37°46′22″ E, 7.V.2022, leg. J. Schmidt, Yeshitla M. (CSCHM).

Paratype: 1 female with same data as holotype and the specimen ID “DH0560” (CSCHM).

Etymology: The new species is named after the village Waber, which is located nearby the type locality.

Description: Body length: 4.7 mm in the holotype, 4.9 mm in the paratype.

Proportions holotype, paratype: PW/HW = 1.47, 1.45; PW/PL = 1.42, 1.43; PW/PBW = 1.21, 1.23; PBW/PAW = 1.30, 1.24; EW/PW = 1.47, 1.48; EL/EW = 1.45, 1.38; EL/AL = 1.76 (holotype).

Colour and microsculpture: As described for *T. basilewskianus*.

Head and prothorax: As described for *T. basilewskianus*.

Pterothorax: Elytra elongate oval in the male and slightly shorter in the female, the sutural angle obtuse in the male and rectangular in the female. All other characters as described for *T. basilewskianus*.

Legs: As described for *T. basilewskianus*.

Aedeagus: Length 1.50 mm. Aedeagus with right side superior in repose. Median lobe elongated, in lateral view about 180° curved in the basal third, straight in the middle, with a very slight ventral convexity before apex; long apical lamella strongly bent upwards and slightly bent backwards, giving a large apical hook. Median lobe tubular in dorsal view, sinusoidally curved from base to apex, the terminal lamella lingulate. Basal bulb of median lobe fairly small, without sagittal aileron. Endophallus entirely covered with sclerotized scales, with long, distally deeply cleft copulatory piece; both tips of the copulatory piece evenly bent diagonally to the length of the median lobe, but only the left tip terminating in a moderately large apical hook (dorsal view). Parameres thin, the left with four, the right with six apical setae.

Differential diagnosis: The new species is externally very similar to *T. basilewskianus* and *T. sebsebei* sp. n., but is easily distinguished from these two species by the median lobe of the aedeagus, which is markedly sinusoidally curved, and by the two-part endophallic copulatory piece. In addition, the endophallus of *T. waberense* sp. n. is more broadly sclerotized than in *T. basilewskianus*, and the median lobe is ventrally straight in the middle but concave in *T. sebsebei* sp. n. For differentiation from other members of the *basilewskianus* subgroup, see the description of these species and the key to species below.

Distribution and geographic variation: Most likely endemic to the mountain range near the village Waber, which extends north from Mt. Choke (Figure 6).

Habitat: As in *T. basilewskianus*.

#### 3.2.7. *Trechus (Abyssinotus) inversus* Schmidt and Faille, sp. n.

**Figure 7:**
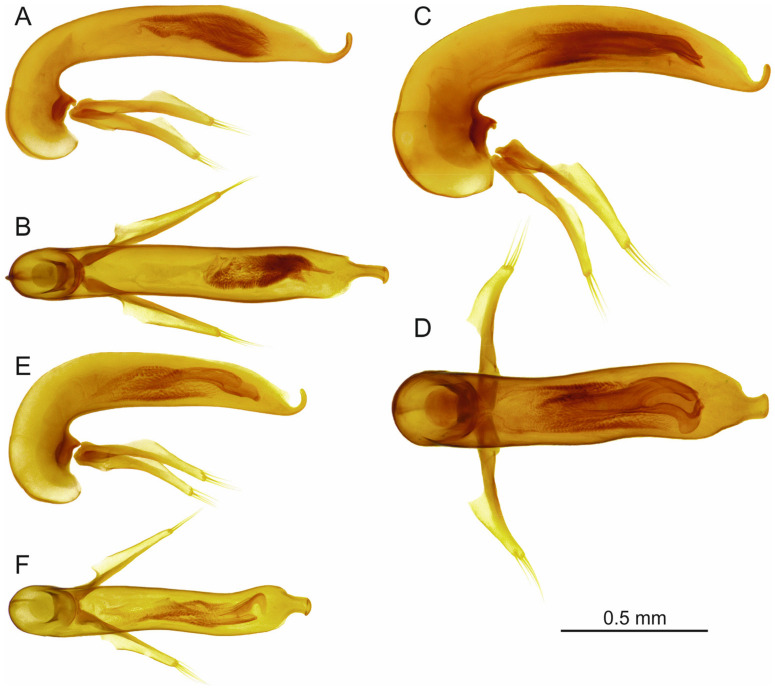
*Trechus* subgenus *Abyssinotus* (*basilewskianus* subgroup), aedeagus, lateral aspect (**A**,**C**,**E**), and dorsal aspect (**B**,**D**,**F**). (**A**,**B**) *T. inversus* sp. n., paratype. (**C**,**D**) *T. infrequens* sp. n., paratype. (**E**,**F**) *T. diversus* sp. n., paratype.

Figure 4D and Figure 7A,B.

Type material: Holotype male, with label data: Ethiopia, Amhara, SW slope Mt. Choke, alt. 3630–3730 m, 10°38′02″ N 37°50′08″ E, 28.II.2022, leg. D. Hauth, J. Schmidt, Yeshitla M., Yitbarek, W. (CSCHM).

Paratype: 6 males, 6 females with same data as holotype (CSCHM, ZSM). A total of 2 females, W slope Mt. Choke, “Shoa Kidaneberet” [Kidanemehret] valley, alt. 3700–3800 m, 10°39′08″ N 37°49′45″ E, 8.V.2022, leg. J. Schmidt, Yeshitla M. (CSCHM).

Etymology: The specific epithet refers to the aedeagus with reverse rotation in repose, which is an extraordinary character state in some species of *Trechus* of northern Ethiopia.

Description: Body length: 3.6–3.9 mm.

Proportions (n = 10): PW/HW = 1.38–1.44 (Ø = 1.41); PW/PL = 1.37–1.42 (Ø = 1.39); PW/PBW = 1.20–1.27 (Ø = 1.24); PBW/PAW = 1.17–1.22 (Ø = 1.20); EW/PW = 1.35–1.40 (Ø = 1.37); EL/EW = 1.46–1.51 (Ø = 1.49); EL/AL = 1.70–1.76 (Ø = 1.72).

Colour: As described for *T. basilewskianus*.

Microsculpture: Head with large, deeply engraved, almost isodiametric sculpticells on the neck, disc, and supraorbital region; smaller sculpticells on the clypeus. Pronotum with large, deeply engraved, slightly transverse sculpticells. Elytral intervals, in both sexes, with moderately deep sculpticells, quite large and slightly more transverse than on the pronotum.

Head: Eyes moderately convexly protruded. All other characters as described for *T. basilewskianus*.

Prothorax, Pterothorax, and legs: As described for *T. basilewskianus*.

Aedeagus: Length 1.20–1.31 mm. Aedeagus with right side superior in repose. Median lobe markedly elongate, quite slender, in lateral view markedly curved in basal third, straight in middle and with slight convexity in apical third, with long, hook-shaped apical lamella bent upwards. Median lobe slender and tubular in dorsal view, with a suggestive sinusoidal curvature from base to apex, the terminal lamella narrowly lingulate. Basal bulb medium-sized, with a suggestion of a sagittal aileron. Endophallus in its apical half entirely covered with sclerotized scales, with a spine-like copulatory piece. Parameres slender, each with four apical setae.

Differential diagnosis: Body size smaller than *T. basilewskianus, T. sebsebei* sp. n. and *T. waberense* sp. n., eyes slightly more protruding. Male genital characters very similar to those of *T. basilewskianus*, but the median lobe of the aedeagus more elongated, the copulatory piece shorter, without an apical hook. In external characters, it is similar to *T. infrequens* sp. n. and *T. diversus* sp. n.; for differentiation from these species see the description of the latter, below. *Trechus inversus* sp. n. differs from the similar species *T. adsuetus* sp. n. and *T. sinuatipenis* sp. n., primarily in having the aedeagus right side superior in repose.

**Figure 8:**
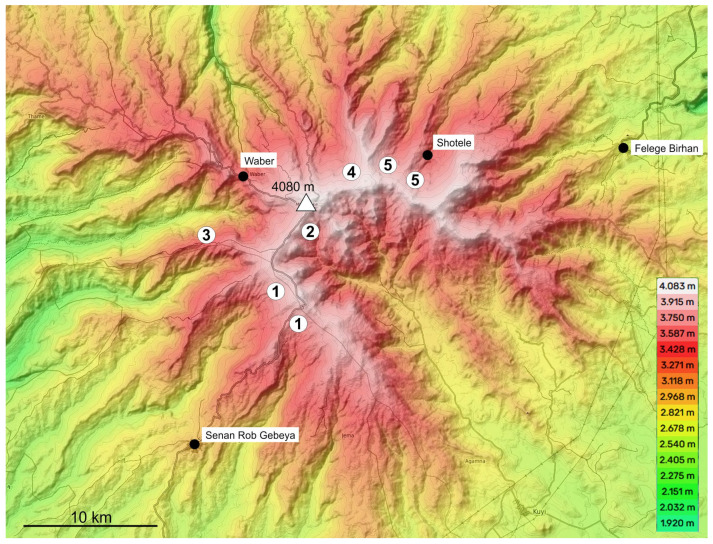
Topographical map of Mt. Choke (highest point marked by a triangle), showing the sampling localities of species in the *T. basilewskianus* subgroup of *Abyssinotus* (white circles). **1**, *T. inversus* sp. n. **2**, *T. infrequens* sp. n. **3**, *T. diversus* sp. n. **4**, *T. adsuetus* sp. n. **5**, *T. sinuatipenis* sp. n. The black circles mark locations of important settlements on the slope of Mt. Choke. Base map from Topographic-map.com.

Distribution: So far, known from two valleys on the south western slope of Mt. Choke (Figure 8).

Habitat: As in *T. basilewskianus*.

#### 3.2.8. *Trechus (Abyssinotus) infrequens* Schmidt and Faille, sp. n.

Figure 4E and Figure 7C,D.

Type material: Holotype male, with label data: Ethiopia, Amhara, Mt. Choke, crater valley, alt. 3780–3900 m, 10°42′12″ N 37°50′58″ E, 27.II.2019, leg. D. Hauth, J. Schmidt, Yeshitla M., Yitbarek, W. (CSCHM).

Paratype: 3 males, 2 females with same data as holotype (CSCHM).

Etymology: The specific epithet refers to the apparent rarity of the species, which presumably has a very small distribution range.

Description: Body length: 3.7–4.0 mm.

Proportions (n = 6): PW/HW = 1.39–1.44 (Ø = 1.41); PW/PL = 1.38–1.41 (Ø = 1.39); PW/PBW = 1.18–1.26 (Ø = 1.22); PBW/PAW = 1.15–1.25 (Ø = 1.20); EW/PW = 1.42–1.51 (Ø = 1.46); EL/EW = 1.39–1.50 (Ø = 1.44); EL/AL = 1.67–1.76 (Ø = 1.73; n = 4).

Colour and microsculpture: As described for *T. basilewskianus*.

Head: Eyes moderately convex and protruding. All other characters as described for *T. basilewskianus*.

Prothorax: As described for *T. basilewskianus*.

Pterothorax: Elytra moderately slender oval. All other characters as described for *T. basilewskianus*.

Legs: As described for *T. basilewskianus*.

Aedeagus: Length 1.25–1.35 mm. Aedeagus with right side superior in repose. Median lobe large and robust, in lateral view markedly curved in the basal third and moderately curved towards the apex; the apical lamella long and hook-like bent upwards. Median lobe broad and tubular in dorsal view, with suggestive sinusoidal curvature from base to apex, the terminal lamella narrowly lingulate. Basal bulb moderately large, without sagittal aileron. Endophallus at apical 2/3 entirely covered with sclerotized scales, the copulatory piece long and broad, longitudinally deeply cleft, the tips directed towards the apex; the two tips bent towards each other (dorsal view), the left of the tips bent in the shape of a rounded hook. Parameres thin, each with four apical setae.

Differential diagnosis: Body smaller than *T. basilewskianus*, *T. sebsebei* sp. n. and *T. waberense* sp. n., eyes slightly more protruding. Male genital characters similar to those of *T. waberense* sp. n., but the median lobe of the aedeagus more robust, ventrally more concave and, in dorsal view, less distinctly sinusoidal. External characters more similar to those of *T. inversus* sp. n., but differing in having smaller and less deeply engraved sculpticells on the pronotal microsculpture; the aedeagus is larger, with a more markedly curved median lobe (lateral view), and with a very large, deeply cleft copulatory piece (with a narrow spine-like piece in *T. inversus* sp. n.). For differentiation from the similar *T. diversus* sp. n. see the description of the latter below. *Trechus infrequens* sp. n. differs from *T. adsuetus* sp. n. and *T. sinuatipenis* sp. n., firstly by having the aedeagus right side superior in repose.

Distribution: So far, known from the western slope of the Mt. Choke crater (Figure 8).

Habitat: As in *T. basilewskianus*.

#### 3.2.9. *Trechus (Abyssinotus) diversus* Schmidt and Faille, sp. n.

Figure 4F and Figure 7E,F.

Type material: Holotype male, with label data: Ethiopia, Amhara, W slope Mt. Choke, alt. 3370 m, 10°38′07″ N 37°45′51″ E, 23.II.2019, leg. D. Hauth, J. Schmidt, Yeshitla M., Yitbarek, W. (CSCHM).

Paratype: 12 males, 18 females with same data as holotype (CSCHM, NHMAA, ZSM).

Etymology: The specific epithet refers to the considerable difference in the characteristics of the male genitalia of this new species compared to the related species and is a further example of the extraordinary diversity of the genital morphology of the *Abyssinotus* species from Mt. Choke.

Description: Body length: 3.6–4.0 mm.

Proportions (n = 10): PW/HW = 1.37–1.45 (Ø = 1.41); PW/PL = 1.36–1.45 (Ø = 1.40); PW/PBW = 1.23–1.30 (Ø = 1.26); PBW/PAW = 1.10–1.20 (Ø = 1.15); EW/PW = 1.42–1.49 (Ø = 1.45); EL/EW = 1.39–1.48 (Ø = 1.44); EL/AL = 1.90–1.98 (Ø = 1.93).

Colour: Dorsal surface medium to dark brown, the head slightly darker than the pronotum and elytra; pronotum in some specimens reddish brown. Mandibles, labrum, and clypeus light brown; palps, antennae, and legs yellowish brown; apical antennomeres slightly darkened in some specimens.

Microsculpture: Head with large, deeply engraved, almost isodiametric sculpticells on the neck, disc, and supraorbital area; smaller sculpticells on clypeus. Pronotum with moderately large, moderately deep and slightly transverse sculpticells. Elytral intervals with moderately deep, fairly large and transverse sculpticells in both sexes.

Head: Eyes slightly convexly protruded. All other characters as described for *T. basilewskianus*.

Prothorax, pterothorax, and legs: As described for *T. basilewskianus*.

Aedeagus: Length 1.02–1.07 mm. Aedeagus with right side superior in repose. Median lobe moderately large and elongated, in lateral view about 180° curved in basal half, almost straight before apex, very slightly bent downwards near apical lamella; latter hook-shaped, bent upwards. In dorsal view, median lobe tubular, slightly sinusoidally curved from base to apex, the terminal lamella lingulate. Basal bulb of median lobe moderately small, without sagittal aileron. Endophallus entirely covered with sclerotized scales, with a long, broad, longitudinally cleft copulatory piece, the tips of which are directed towards the apex; the two tips of the piece extending in a flat arc from the centre of the median lobe to the apical ostium; the right tip ends in a large apical hook (dorsal view). Parameres thin, each with four or five apical setae.

Differential diagnosis: Externally very similar to *T. infrequens* sp. n. and *T. inversus* sp. n., but differing by a smaller aedeagus, with a distinctly sinusoidally curved median lobe (dorsal view; straight or almost straight in *T. inversus* sp. n. and *T. infrequens* sp. n.), median lobe thinner (robust in *T. infrequens* sp. n.), and endophallic copulatory piece long and broad (narrow in *T. inversus* sp. n.), with the right apical tip of the piece hook-like curved (whereas the left apical tip hook-like curved in *T. infrequens* sp. n.). *Trechus diversus* sp. n. differs from the likewise very similar species *T. adsuetus* sp. n. and *T. sinuatipenis* sp. n., primarily, by the aedeagus with right side superior in repose.

Distribution: Probably endemic to a single mountain range extending from Choke crater to the west (Figure 8).

Habitat: As in *T. basilewskianus*.

#### 3.2.10. *Trechus (Abyssinotus) adsuetus* Schmidt and Faille, sp. n.

Figure 9A–C.

Type material: Holotype male, with label data: Ethiopia, Amhara, N slope Mt. Choke, alt. 3750–3850 m, 10°43′51″ N 37°52′15″ E, 9.V.2022, leg. J. Schmidt, Yeshitla M. (CSCHM).

Paratype: 4 males, 1 female with same data as holotype (CSCHM).

Etymology: The specific epithet is derived from the Latin adjective ‘adsuetus’ = regular, standard, and refers to the fact that in this species, the aedeagus is in the ‘normal’ position with respect to Trechinae, with left side superior in repose.

Description: Body length: 4.1–4.5 mm.

Proportions (n = 6): PW/HW = 1.38–1.42 (Ø = 1.40); PW/PL = 1.36–1.43 (Ø = 1.40); PW/PBW = 1.15–1.25 (Ø = 1.22); PBW/PAW = 1.18–1.28 (Ø = 1.23); EW/PW = 1.40–1.49 (Ø = 1.44); EL/EW = 1.44–1.50 (Ø = 1.48); EL/AL = 1.57–1.61 (Ø = 1.59).

Colour and microsculpture: As described for *T. basilewskianus*.

Head: Eyes slightly convexly protruded. All other characters as described for *T. basilewskianus*.

Prothorax, pterothorax, and legs: As described for *T. basilewskianus*.

Aedeagus: Length 1.39–1.52 mm. Aedeagus with left side superior in repose. Median lobe large and elongated, in lateral view markedly curved in the basal third, straight towards the apex, with a long, hook-like and upwards curved apical lamella. Median lobe in dorsal view slender and tubular, suggestively sinusoidal from base to apex, the terminal lamella slender lingulate. Basal bulb of median lobe moderately small, without sagittal aileron. Endophallus entirely covered with sclerotized scales, with a long, broad copulatory piece, in dorsal view slightly curved, longitudinally cleft, the tips of which are directed apicad; the left tip is longer than the right and ends in a sinusoidally curved ribbon-like structure. Parameres slender, each with four apical setae.

Differential diagnosis: This new species differs from all other species of the *basilewskianus* subgroup of *Abyssinotus*, with the exception of *T. sinuatipenis* sp. n., in having the left side of the aedeagus superior in repose. Moreover, it differs from all species in this group, including *T. sinuatipenis* sp. n., by the remarkable shape of the copulatory piece with its left apical ribbon-like end, sinusoidally curved at the apex. For other differences from *T. sinuatipenis* sp. n., see the description of the latter species below.

Distribution: Probably endemic to the northern slope of Mt. Choke (Figure 8).

Habitat: As in *T. basilewskianus* (photo of the habitat see Figure 3)

#### 3.2.11. *Trechus (Abyssinotus) sinuatipenis* Schmidt and Faille, sp. n.

Figure 9D–F.

Type material: Holotype male, with label data: Ethiopia, Amhara, NE slope Mt. Choke, alt. 3700–3880 m, 10°42′56″ N 37°55′16″ E, 4.V.2022, leg. J. Schmidt, Yeshitla M. (CSCHM).

Paratype: 22 males, 12 females with same data as holotype (CAF, CSCHM, NHMAA, ZSM). A total of 2 males, NE slope Mt. Choke, above Gumadur [Shotele], alt. 3750–3850 m, 10°44′10″ N 37°53′48″ E, 5.V.2022, leg. J. Schmidt, Yeshitla M. (CSCHM).

Etymology: The specific epithet refers to the median lobe of the aedeagus of the new species, which has a marked sinusoidal curvature.

Description: Body length: 3.4–3.9 mm.

Proportions (n = 10): PW/HW = 1.35–1.44 (Ø = 1.39); PW/PL = 1.35–1.43 (Ø = 1.40); PW/PBW = 1.19–1.25 (Ø = 1.22); PBW/PAW = 1.14–1.24 (Ø = 1.20); EW/PW = 1.41–1.47 (Ø = 1.44); EL/EW = 1.42–1.47 (Ø = 1.44); EL/AL = 1.51–1.60 (Ø = 1.56).

Colour and microsculpture: As described for *T. basilewskianus*.

Head: Eyes slightly convexly protruded. In all other characters as described for *T. basilewskianus*.

Prothorax, pterothorax, and legs: As described for *T. basilewskianus*.

Aedeagus: Length 1.23–1.30 mm. Aedeagus with left side superior in repose. Median lobe large and elongated, in lateral view markedly curved in basal third, with ventral side straight in middle, with a distinct concavity before apex, and with apical lamella long and hook-shaped curved upwards. In dorsal view, median lobe slender, tubular, markedly sinusoidally curved from base to apex, the terminal lamella slender lingulate. Basal bulb of median lobe moderately small, without sagittal aileron. Median part of the endophallus covered with small, sclerotized scales; copulatory piece long, apically deeply cleft, in lateral view almost straight, in dorsal view with both parts of the piece rod-shaped and evenly bent to the left, with tips simply rounded. Parameres thin, each with four apical setae.

Differential diagnosis: This new species differs from all other species of the *basilewskianus* subgroup of *Abyssinotus*, with the exception of *T. adsuetus* sp. n., in having the aedeagus with the left side superior in repose. In addition, it differs from all species in this group, including *T. adsuetus* sp. n., in the shape of the two apices of the longitudinally cleft copulatory piece, which are evenly curved and rod-shaped (dorsal view). The new species also differs from *T. adsuetus* sp. n. in the median lobe of the aedeagus, which is markedly sinusoidally curved when viewed dorsally.

Distribution: Probably endemic to the north eastern slope of Mt. Choke (Figure 8).

Habitat: As in *T. basilewskianus*.

### 3.3. Key to Species of Trechus Subgenus Abyssinotus from Mt. Choke

**1** Body length ≥ 5.2 mm… *T. chokensis* Pawlowski, *T. sabae* (Quéinnec and Ollivier) and species of the *dimorphicus* and *gigas* subgroups, see key to species in [3,4].

**-** Body length < 5.2 mm…**2**

**2** Tiny (body length 2.0–2.4 mm), microphthalmic species with compound eyes about half as long as tempora. Entire body yellowish brown. Male protarsomeres not dilated… ***T. hauthi* Schmidt and Faille**

**-** Compound eyes as long as or longer than tempora. Body reddish to dark brown. At least basal protarsomere of male dilated…**3**

**3** Protarsomere 1 of male dilated…**4**

**-** Protarsomeres 1 and 2 of male dilated…**8**

**4** Pronotal basolateral angles markedly large, rectangular or acute. Aedeagus with right side superior in repose. Endophallus without marked sclerotization…**5**

**-** Pronotal basolateral angles small, obtuse, or rounded. Aedeagus with left side superior in repose. Endophallus +/− markedly sclerotized…**6**

**5** Shape of pronotum as in *Zabrus*, with sides not clearly converging towards the base, although sinuate. Aedeagal median lobe elongate, parameres slender… ***T. lobeliae* (Quéinnec and Ollivier)**

**-** Sides of pronotum clearly convergent towards the base. Aedeagal median lobe markedly curved, parameres short … ***T. inermus* sp. n.**

**6** Elytra with second discal seta absent… ***T. amharicus* Ortuño and Novoa**

**-** Elytra with second discal seta present… **7**

**7** First protarsomere of male moderately dilated with long apical tooth at inner margin. Aedeagal median lobe with a well sclerotized, hook-shaped apical lamella bent upwards… ***T. afroalpinus* (Quéinnec and Ollivier)**

**-** First protarsomere of male very slightly dilated with a short apical tooth. Aedeagal median lobe with a very weakly sclerotized apical lamella, with rounded tip… ***T. abyssinicus* (Quéinnec and Ollivier)**

**8** Pronotal basolateral angles large and rectangular …***T. chokensis* Pawlowski**

**-** Pronotal basolateral angles small and obtuse… **9**

**9** Body and appendages stocky, elytra shorter (EL/EW < 1.36), third antennomere shorter than second, right mandible with three distinct denticles, without recognisable separation of premolar and retinaculum… *niloticus* subgroup, see key to species in Schmidt et al. [12]

**-** Body and appendages more slender, elytra longer (EL/EW > 1.37), third antennomere longer than second, right mandible with sharp premolar but indistinctly bifid retinaculum, and with well-separated premolar and retinaculum (the *basilwskianus* subgroup)… **10**

**9** Aedeagus with left side superior in repose… **11**

**-** Aedeagus with right side superior in repose … **12**

**11** Aedeagus, in dorsal view, markedly sinusoidally curved from base to apex. Shape of the two apices of the copulatory piece nearly identical, evenly curved and rod-shaped… ***T. sinuatipenis* sp. n.**

**-** Aedeagus, in dorsal view, almost straight. Shape of the two apices of the copulatory piece markedly different, with the left one ribbon-shaped and sinusoidally curved… ***T. adsuetus* sp. n.**

**12** Copulatory piece deeply cleft in distal part, giving two well-separated ends in the shape of a rod or a ribbon… **13**

**-** Copulatory piece not cleft distally… **15**

**13** Body size > 4.6 mm… ***T. waberense* sp. n.**

**-** Body size < 4.1 mm … **14**

**14** Aedeagal median lobe slender, copulatory piece with right apical tip hook-shaped… ***T. diversus* sp. n.**

**-** Aedeagal median lobe robust, copulatory piece with left apical tip hook-shaped… ***T. infrequens* sp. n.**

**15** Median lobe of aedeagus, in lateral view, with ventral margin markedly concave along entire length, the terminal lamella curved upwards with tip distinctly curved backwards (*T. sebsebei* sp. n.)… **16**

**-** Median lobe of aedeagus, in lateral view, with ventral margin slightly concave, straight or slightly convex; terminal lamella curved upwards with tip not curved backwards … **18**

**16** Length of aedeagal median lobe > 1.7 mm… ***T. sebsebei extremus* ssp. n.**

**-** Length of aedeagal median lobe < 1.61 mm… **17**

**17** Length of aedeagal median lobe 1.45–1.60 mm… ***T. sebsebei* sp. n. (s. str.)**

**-** Length of aedeagal median lobe 1.32–1.42 mm… ***T. sebsebei curvipenis* ssp. n.**

**18** Copulatory piece in the form of a narrow spine… ***T. inversus* sp. n.**

**-** Rod-shaped copulatory piece with hooked apex… **19**

**19** Aedeagal median lobe shorter compared to the body length (EL/AL > 1.7)… ***T. basilewskianus* Geginat (s. str.)**

**-** Aedeagal median lobe longer compared to the body length (EL/AL < 1.7)… ***T. basilewskianus extendipenis* ssp. n.**

## 4. Discussion

The two species groups revised in this paper most likely represent independent evolutionary lineages within *Abyssinotus* and have, therefore, been designated as subgroups of the subgenus. As other geographically separate high montane habitats in northern Ethiopia, such as Mt. Guna and the Simien Mts., have hardly been studied to date, it remains unclear whether the *T. basilewskianus* subgroup and the *T. lobeliae* subgroup represent lineages endemic to Mt. Choke or whether they also occur on the volcanoes mentioned.

We consider that the markedly long, upwards curved terminal lamella of the aedeagal median lobe is a synapomorphy of the *T. basilewskianus* subgroup. The monophyly hypothesis is also supported by the extreme external similarity of the species in this group, which are remarkable within the *Trechus* fauna of Ethiopia because of their slender shape and *Agonum*-like body, and by the great similarities in the structure of the median lobe of the aedeagus and the endophallus. With regard to the *T. lobeliae* subgroup, we assume that two characters states are synapomorphies, namely i) the aedeagus with its right side superior in repose and ii) the completely membranous endophallus, without copulatory piece and sclerotized scales. While the latter is unique within the Ethiopian *Trechus*, the first character state is also developed in most species of the *T. basilewskianus* subgroup. However, previously published molecular phylogenetic data lead us to suggest that the character state ‘aedeagus right side superior in repose’ was developed twice independently in the evolution of *Abyssinotus* (Figure 10). Faille et al. [8] show that *T. lobeliae* is the sister species of a clade including *T. hauthi* Schmidt and Faille, 2024, the *T. abyssinicus* subgroup (=*Afrotrechus* Quéinnec and Ollivier, 2021), the *T. niloticus* subgroup (=*Nilotrechus* Quéinnec and Ollivier, 2021) and the *T. basilewskianus* subgroup, the latter two being sister taxa but with low support. On the basis of these data, inverse rotation of the aedeagus most likely occurred in the stem group of the *T. lobeliae* subgroup, and a second time either in the stem group of the *T. basilewskianus* subgroup, or during a later phase of the evolution of the group. To date, no molecular data are available to provide information on the diversification and morphological evolution of the *T. basilewskianus* subgroup. It is, therefore, not possible to say whether the two species characterised by an aedeagus in the normal position, *T. adsuetus* sp. n. and *T. sinuatipenis* sp. n., are the most basal species in this group and whether the inverse rotation of the aedeagus only evolved in a terminal clade of the group, or whether the normal state in these species appeared secondarily, i.e., whether it is an apomorphy.

The aedeagus of the Carabidae is a morphological peculiarity among Coleoptera due to its position, which is rotated 90° on the right side of the abdomen, with the left side superior in repose [13]. However, this position does not appear to be strictly fixed, and the inverted state was more commonly observed in species and species groups of the ground beetle subfamily Harpalinae. *Harpalus rufipes* (DeGeer, 1774) is very widespread in the Palearctic region [19]. In this species, the aedeagus is actually found with left side superior in repose, but Jeannel [20] found that in some specimens collected in Bretagne, the aedeagus is found with the right side superior in repose. On the other hand, an inverted position of the aedeagus is characteristic for all species in the Pterostichini subtribe Caelostomina [21,22], the Ctenodactylini [13], and the Sphodrini subtribe Pristosiina [23,24]. Remarkably, there is another species of the tribe Sphodrini with an inverted aedeagus, *Calathus ovipennis* Putzeys, 1873, which is the only known species with this feature in the megadiverse subtribe Calathina [25].

We have found no reference in the literature to the description of an inverse position of the aedeagus in a species or species group of the so-called “basal-grade” carabids [26] but several examples in species of “middle-grade” carabids [27]. Roig-Juñent [28] found that some species of the Broscini genus *Promecoderus* Dejean, 1829 exhibit an inversion of the aedeagus. Liebherr and Will [15] present an overview to species of the tribe Moriomorphini with inverted male genitalia. Within the specious genus *Mecyclothorax*, only *M. storeyi* Moore, 1984, monomorphically includes males with inverted genitalia [29,30], but this character state varied from 0 to 58% in West Australian populations of *M. punctipennis* (MacLeay, 1871) [15]. On the other hand, inverted male genitalia are found in all species of the South American moriomorphine genus *Tropopterus* Solier, 1849, and this character state is considered synapomorphy of the group [31,32]. To date, monomorphic species characterised by inverted male genitalia were unknown for the subfamily Trechinae. However, the rotation of male genitalia can be assumed from the observation of mirrored aedeagi in single individuals of Trechinae species for which an aedeagus in ‘normal’ position is the usual condition. This case was documented by Maddison [33] in a specimen of the *Bembidion kuprianovi* Mannerheim, 1843 complex. Donabauer in [33] noted that he observed mirrored aedeagi in a few individuals of two *Trechus* populations from Turkey among others with normal ones. Unfortunately, this latter example from trechine beetles was never described in detail and, therefore, the morphological expression of chiral polymorphism in male genitalia of this group of ground beetles is unknown so far.

**Figure 9:**
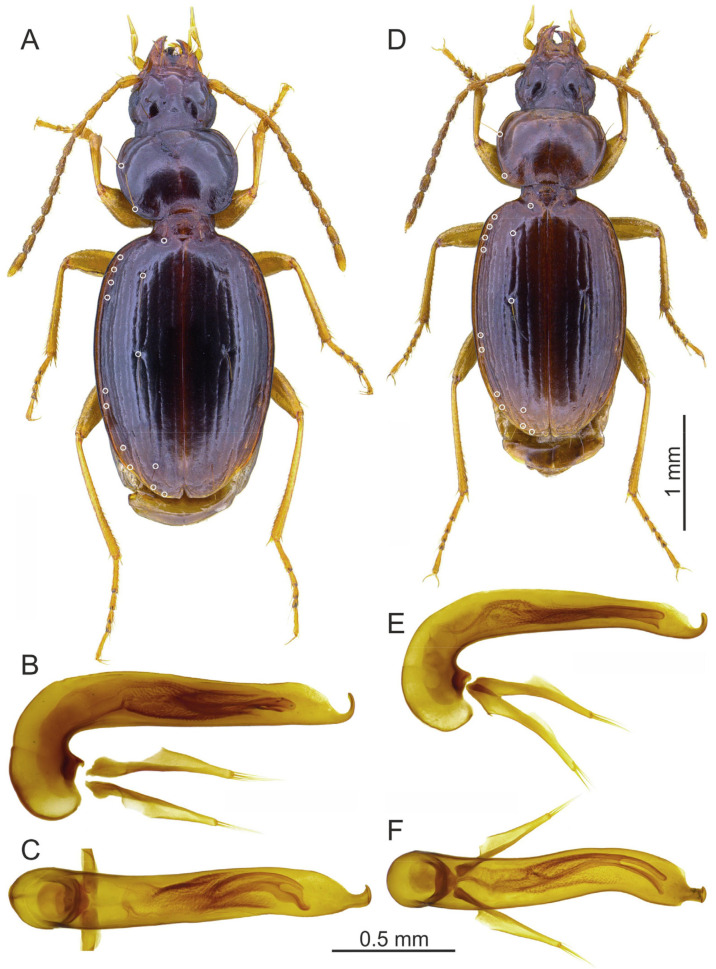
*Trechus* subgenus *Abyssinotus* (*basilewskianus* subgroup) male specimens, dorsal aspect of the body (**A**,**D**), aedeagus in lateral view (**B**,**E**) and dorsal view (**C**,**F**). (**A**–**C**) *T. adsuetus* sp. n., paratypes. (**D**–**F**) *T. sinuatipenis* sp. n., paratypes. The white circles point to the insertions of the pronotal lateral setae, parascutellar seta, elytral discal setae, and setae of the umbilicate series.

**Figure 10:**
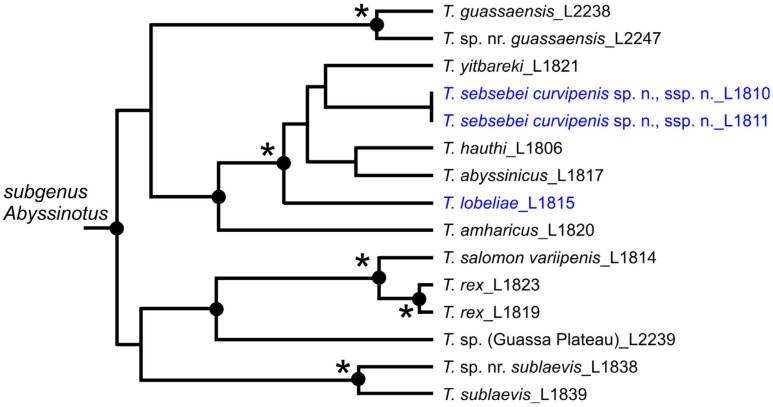
*Trechus* (*Abyssinotus*) subtree of the molecular phylogeny of trechine beetles by Faille et al. [8], modified (see that paper for details; taxon names have been changed according to most recent taxonomy [4,5,12]). Black circles and stars at branch nodes refer to posterior probabilities ≥ 0.98 and bootstrap values > 75, respectively. Species of the *T. lobelia* subgroup and the *T. basilewskianus* subgroup are highlighted blue.

**Figure 11:**
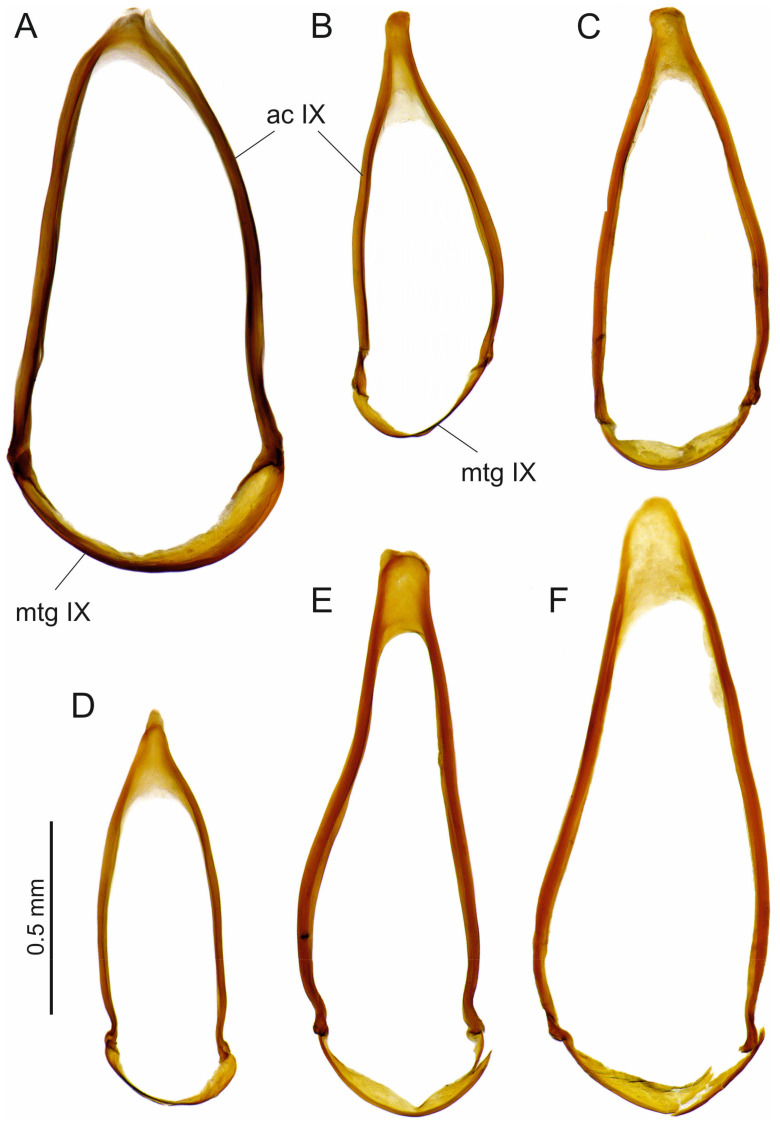
*Trechus* subgenus *Abyssinotus*, abdominal segment IX of males, dorsal aspect. (**A**–**C**) Species with aedeagus left side up in repose. (**D**–**F**) Species with aedeagus right side up in repose. (**A**) *T. salomon*. (**B**) *T. amharicus*. (**C**) *T. sinuatipenis* sp. n. (**D**) *T. lobeliae*. (**E**) *T. sebsebei* sp. n. (s. str.). (**F**) *T. sebsebei extremus* ssp. n. Abbreviations: mtg, metatergite; ac, antecosta.

**Figure 12:**
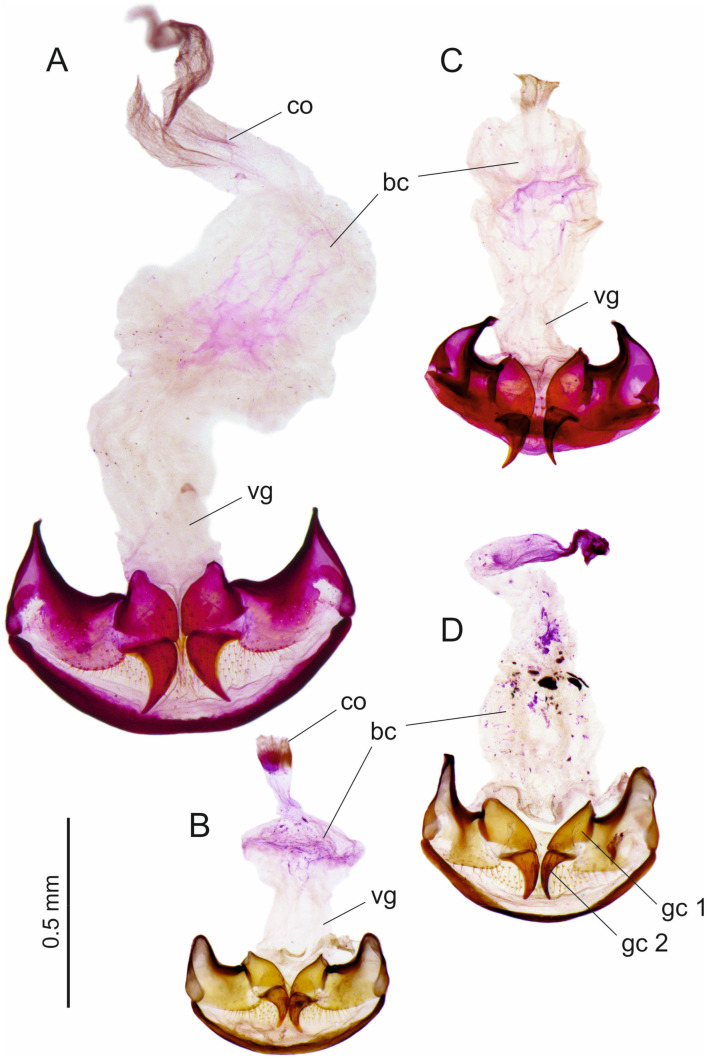
*Trechus* subgenus *Abyssinotus*, female reproductive tract, ventral aspect. (**A**,**B**) Species with aedeagus left side up in repose. (**C**,**D**) Species with aedeagus right side up in repose. (**A**) *T. salomon*. (**B**) *T. amharicus*. (**C**) *T. lobelia*. (**D**) *T. sebsebei* sp. n. (s. str.). Abbreviations: bc, bursa copulatrix; co, common oviduct; gc 1, basal gonocoxite; gc 2, apical gonocoxite; vg, vagina.

In the Ethiopian *Trechus* species we cannot prove any mirroring of the male genitalia. In the subgenus *Abyssinotus*, such detection is difficult due to the almost symmetrical morphology of the parameres, the ventral or dorsal position of the two ostia, and the sac-shaped structure of the endophallus with the central position of the copulatory piece, if such a piece is developed at all. Consequently, aedeagi of monomorphic ‘normal species’ and those of monomorphic species with inverted genitalia seem identical in the general genital structures (see Figure 5, Figure 7 and Figure 9). The same applies to species of many other groups of Trechini, which have practically lateral-symmetric male genitalia, e.g., from the genera *Aepus* Samouelle, 1819, *Anophthalmus* Sturm, 1844, *Baehria* Schmidt and Faille 2023, *Duvalius* Delarouzée, 1859, *Perileptus* Schaum 1860, *Trechisibus* Motschulsky, 1863, *Trechodes* Blackburn, 1901, and many others [8,34,35]. We also examined the abdominal segment IX of the male (‘ring segment’ that surrounds the aedeagus in repose) and the genital tract of the female in several species of the Ethiopian *Trechus* but again found no evidence of morphological changes due to mirroring of the male genitalia (examples are shown in Figure 11 and Figure 12). According to our preliminary results, interspecific differences in the shape of abdominal segment IX cannot be derived from the position of the aedeagus. The morphological variability in this segment between subspecies of a species (e.g., *T. s. sebsebei* and *T. s. extremus*) was found to be nearly as high as between species that have a different position of the aedeagus (left or right side up in repose; see Figure 11). The female genital tract has a simple sac-like bursa copulatrix, which does not show any sclerotized spermatheca in the Ethiopian species (Figure 12). This finding is largely consistent with studies in other *Trechus* lineages [36,37]. We have so far been unable to find any evidence as to whether mirroring of the aedeagus has a recognisable effect on the morphology of the female genital tract in the Ethiopian *Trechus*.

Ethiopian *Trechus,* thus, provide the first examples of the existence of monomorphic species with inverted male genitalia in the tribe Trechini. It is therefore particularly surprising that this morphological change likely evolved twice independently within the *Trechus* subgenus *Abyssinotus*. This demonstrates once again the particular importance of the fauna of the East African highlands for the study of evolution in ground beetles. Clearly, the towering volcanic mountains are hotspots for speciation and morphological radiation of groups of cold-adapted species [3,4]. In fact, the Afroalpine fauna of Ethiopia is clearly dominated in a number of species by only two groups of species native to the Palearctic region, *Trechus* and *Calathus* Bonelli, 1810 [1]. From Mt. Choke alone, with the new taxa described here, a total of 28 species and 4 additional subspecies of the *Trechus* subgenus *Abyssinotus* have been described, all endemic to this volcano [1,4,5,12]. Furthermore, all these species, although very closely related, are characterised by extreme interspecific variability in their morphological characteristics, which cannot be observed in this dimension anywhere else in the distribution range of the megadiverse genus *Trechus* s. l. [4]. The demonstration of the development of genitalia inversion in two lineages of *Abyssinotus* is further evidence of the special position of the Afro-alpine fauna of *Trechus* for evolutionary studies, and should stimulate further intensive investigations, in particular using molecular methods, but also an even greater expansion of field research. It is very likely that many other, as yet unknown, species are present in the Ethiopian highlands, whose character traits may provide a great deal of other interesting insights into the morphological evolution of the group.

## Data Availability

All research data supporting the present work have been cited in full.
